# Uniform and accelerated degradation of pure iron patterned by Pt disc arrays

**DOI:** 10.1038/srep23627

**Published:** 2016-04-01

**Authors:** Tao Huang, Yufeng Zheng

**Affiliations:** 1 State Key Laboratory for Turbulence and Complex System and Department of Materials Science and Engineering, College of Engineering, Peking University, Beijing 100871, China

## Abstract

Pure iron has been confirmed as a promising biodegradable metal. However, the degradation rate of pure iron should be accelerated to meet the clinical requirements. In this work, two different designs of platinum disc arrays, including sizes of Φ20 μm × S5 μm and Φ4 μm × S4 μm, have been coated on the surface of pure iron. Corrosion tests showed the platinum discs formed plenty of galvanic cells with the iron matrix which significantly accelerated the degradation of pure iron. Simultaneously, due to the designability of the shape, size as well as distribution of Pt discs, the degradation rate as well as degradation uniformity of pure iron can be effectively controlled by coating with platinum discs. The cytotoxicity test results unveiled that Pt discs patterned pure iron exhibited almost no toxicity to human umbilical vein endothelial cells, but a significant inhibition on proliferation of vascular smooth muscle cells. In addition, the hemolysis rate of Pt discs patterned pure iron was lower than 1%. Moreover, Pt discs also effectively reduced the number of adhered platelets. All these results indicated that Pt discs patterning is an effective way to accelerate degradation and improve biocompatibility of pure iron.

Biodegradable stents are considered to be the next generation of stents^1^, which can effectively avoid late stent thrombosis that happens frequently among permanent stents. Iron-based material^2^,^3^,^4^,^5^,^6^,^7^ has been considered to be one of the most potential candidates for biodegradable stent applications.

Iron is an essential nutrient element in human body which plays a vital role in many biochemical reactions, such as induction and transportation of oxygen, electron transferring, catalyst, and so forth^8^. Besides, iron also has good mechanical properties as well as good biocompatibility^2^,^9^,^10^, which are close to that of 316L stainless steel. In 2001, pure iron was first implanted into New Zealand white rabbits and its safety as stent material was verified^11^. From then on, the biosafety of pure iron stents was further confirmed through a series of both *in vitro* and *in vivo* investigations^9^,^12^,^13^,^14^,^15^,^16^. Nevertheless, too slow degradation of pure iron was found which cannot meet the clinical requirement. Moreover, localized pitting corrosion was found to be the main corrosion mode of pure iron in physiological environment, which may cause early fracture of stent. Therefore, iron based materials with faster degradation and more uniform corrosion modes need to be developed.

Up to now, numerous methods have been tried to enhance mechanical properties and corrosion rate of pure iron, such as alloying^3^,^14^,^17^,^18^,^19^, compositing as well as new preparation techniques^20^,^21^,^22^,^23^. Some of these methods sped up the degradation of pure iron, but not enough. Researches on surface modification of pure iron have also been reported, such as Fe-O thin film^24^, calcium zinc phosphate coating^25^, lanthanum ion implanting^26^, and ion nitriding^27^. Most of these previous researches on surface modification of pure iron significantly improved biocompatibility of pure iron, but in the meantime, the corrosion resistance was enhanced. Therefore, these methods are inconsistent with the goal of making pure iron more suitable for biodegradable implant applications.

Surface patterning has been demonstrated as an effective way to adjust the adhesion, stretch and proliferation of cells, thereby improving the interaction between implants and the host^28^,^29^,^30^. In the field of biomedical metallic materials, surface patterning was frequently adopted to regulate cells behavior on the implants made of pure titanium and titanium oxide^31^,^32^. However, using surface patterning to control corrosion behavior of metallic materials has not been reported previously.

In this work, platinum disc arrays were prepared on the surface of pure iron through photolithography and electron beam evaporation. Platinum has been confirmed to be a material with excellent hemocompatibility^33^. And because of its high corrosion potential, platinum discs can be introduced to accelerate degradation rate of pure iron matrix through forming galvanic cells. Furthermore, the corrosion rate and distribution can be effectively controlled via designing the size, shape and distribution of platinum discs.

## Results

### Microstructure and chemical characterization of Pt discs patterned pure iron

**Figure 1:**
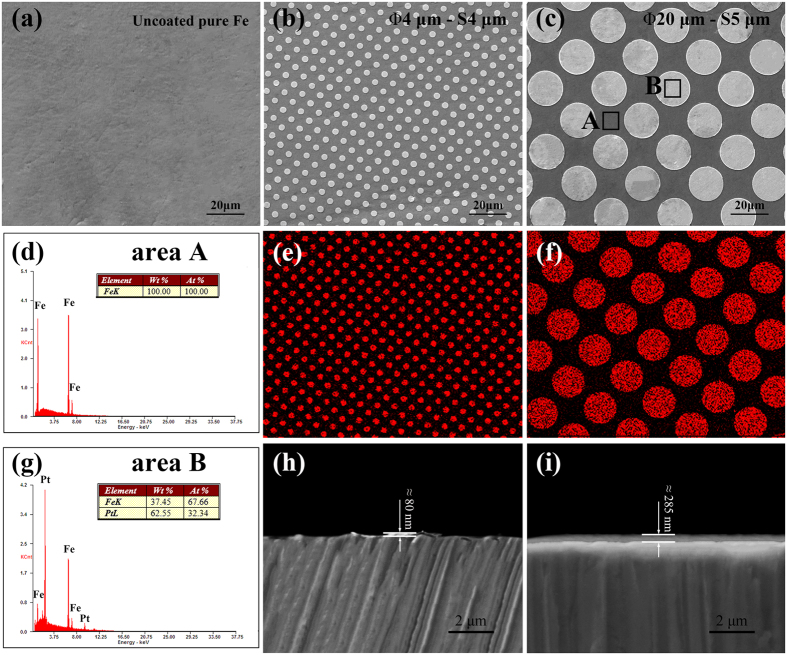
Microstructure of Pt discs patterned pure iron: (**a**) uncoated pure iron, (**b**) Φ4 μm × S4 μm and (**c**) Φ20 μm × S5 μm Pt discs patterned pure iron, (**d**) and (**g**) are energy spectrum analysis related to area A and B respectively, (**e**) and (**f**) are energy spectrum plane scanning analysis of Pt, (**h**) and (**i**) are the cross sections of Φ4 μm × S4 μm and Φ20 μm × S5 μm patterned pure iron, respectively.

Figure 1 shows the ESEM images and the EDS analysis of Pt discs patterned pure iron, with uncoated pure iron as control. In this work, two different designs of platinum disc arrays including sizes of Φ20 μm × S5 μm (Φ20 μm represents that the diameter of the platinum disc is 20 μm, S5 μm means that the space between two nearest platinum discs is 5 μm) and Φ4 μm × S4 μm were prepared. According to the cross section images, the thickness of Pt disc with size of Φ4 μm × S4 μm is about 80 nm, while that of the Φ20 μm × S5 μm one is approximately 285 nm.

### Electrochemical corrosion behavior of Pt discs patterned pure iron

**Figure 2:**
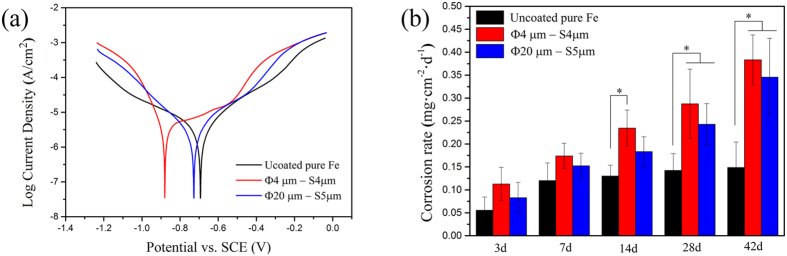
(**a**) potentiodynamic polarization curves of Pt discs patterned pure iron in Hank’s solution, (**b**) corrosion rates calculated from weight loss of samples after static immersion in Hank’s solution. ^*^represents p < 0.05.

Figure 2(a) shows the potentiodynamic polarization curves of Pt disc arrays patterned pure iron in Hank’s solution, and the mean electrochemical parameters are listed on Table 1. Comparing to those of pure iron, patterning by Pt discs significantly decreased the corrosion potential and increased the corrosion current density, indicating greater tendency to be corroded. Among experimental samples, the group with size of Φ4 μm × S4 μm exhibited the highest corrosion tendency.

### Static immersion corrosion behavior of Pt discs patterned pure iron

**Figure 3:**
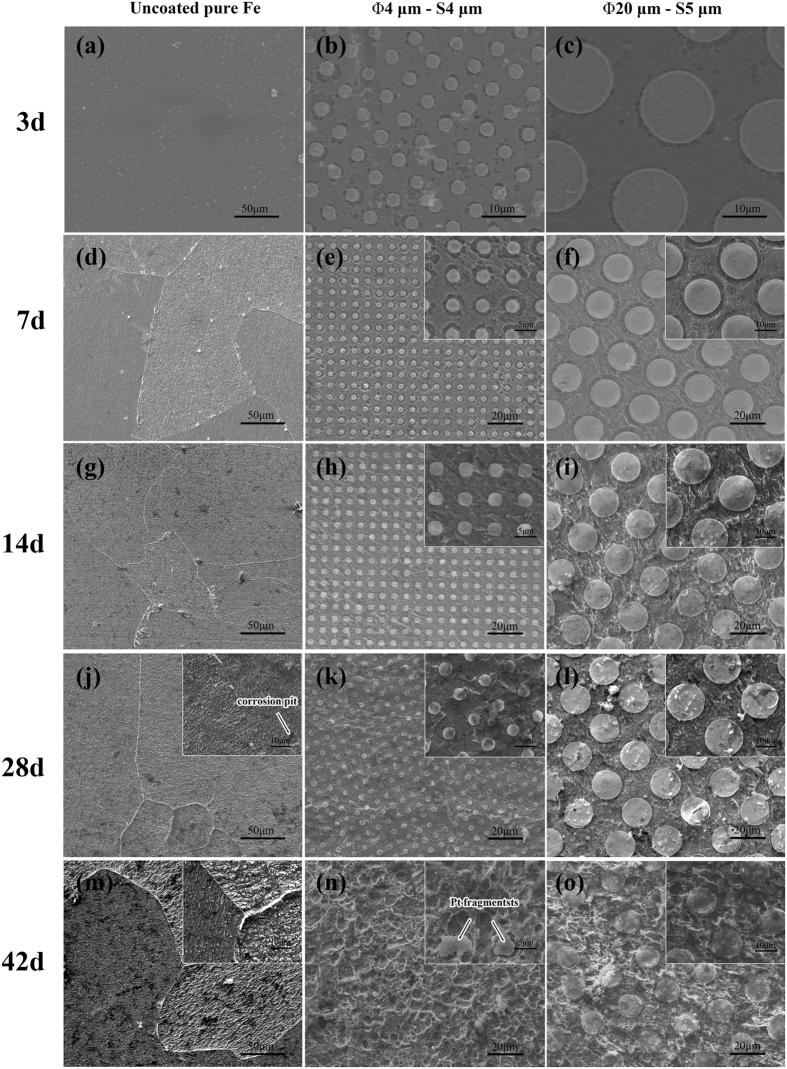
SEM images of samples’ surface morphology after statically immersed in Hank’s solution for 3, 7, 14, 28 and 42 days.

Figure 3 shows the surface morphology of Pt discs patterned pure iron samples after 3, 7, 14, 28 and 42 days static immersion in Hank’s solution. As can be seen from Fig. 3:

1. After 3 days immersion, the surface of pure iron kept intact, only a few deposited salts were observed. As for Pt discs patterned pure iron samples, corrosion happened first on the iron matrix which closely surrounded Pt discs, and this phenomenon was more obvious on the Φ4 μm × S4 μm samples.
2. After 7 days immersion, grain boundary could be observed clearly on the surface of uncoated pure iron. On the surface of Pt discs patterned pure iron, the corrosion range continued spreading outward.
3. After 14 days immersion, the corrosion of uncoated pure iron relatively deepened. On the surface of Pt discs patterned pure iron, the corrosion extended to the whole exposed pure iron matrix. In a macro perspective, the corrosion was uniform.
4. After 28 days immersion, on the surface of uncoated pure iron, it can be clearly observed that corrosion directions varied on grains with different orientations. Corrosion on the surface of Pt discs patterned pure iron started penetrating into the iron matrix covered by Pt discs.
5. After 42 days immersion, localized corrosion pits began emerging on the surface of uncoated pure iron. Pt discs with size of Φ4 μm × S4 μm fell off from the pure iron matrix and corresponding corrosion pits remained *in situ*. On the surface of Pt discs patterned pure iron with size of Φ20 μm × S5 μm, corrosion of pure iron matrix continued stretching to the center under Pt discs.

Figure 2(b) shows the corrosion rates calculated from weight loss after samples immersed in Hank’s solution for 3, 7, 14, 28 and 42 days, respectively. Pt discs patterned pure iron kept faster corrosion rate than that of uncoated one through the whole experimental period, and the Pt discs patterned pure iron with size of Φ4 μm × S4 μm exhibited the fastest corrosion. Corrosion rates of all samples increased with time.

### Cytotoxicity of Pt discs patterned pure iron

**Figure 4:**
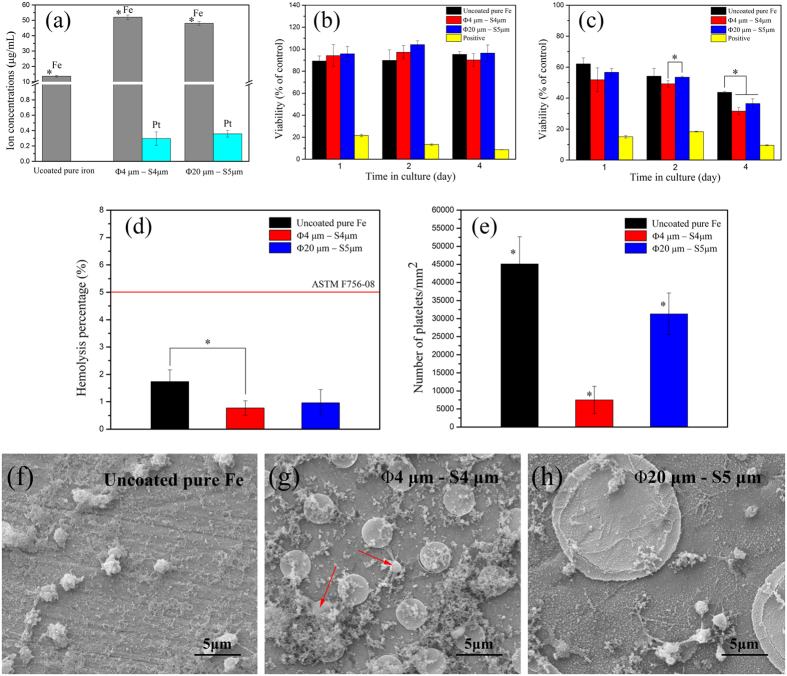
(**a**) ion concentration in experimental materials’ extraction mediums, cell viability of (**b**) EA. Hy-926 and (**c**) VSMC after cultured in extraction mediums and positive control for 1, 2 and 4 days, (**d**) hemolysis of Pt discs patterned pure iron, (**e**) the number of adhered platelets per unit area on the surface of samples, (**f–h**) are morphologies of platelets adhered on the surface of uncoated pure iron and Pt discs patterned pure iron. ^*^represents p < 0.05.

Figure 4(a–c) illustrates the ion concentrations of extractions (a) and the cell viabilities of (b) human umbilical vein endothelial cells EA. hy-926 and (c) human vascular smooth musle cells (VSMC) after 1, 2 and 4 days incubation in experimental materials extraction mediums. According to Fig. 4(a), the order of iron ion concentration in extraction mediums was: Φ4 μm × S4 μm (52.494 ± 1.4374 μg∙mL^−1^) > Φ20 μm × S5 μm (48.784 ± 1.215 μg∙mL^−1^) > uncoated pure iron (13.601 ± 0.5482 μg∙mL^−1^), which matched well with the corrosion results of static immersion tests in Hank’s solution. The corrosion rate of Pt discs patterned pure iron in DMEM was about 4 times faster than that of uncoated pure iron. Furthermore, because of the high chemical stability of Pt, Pt ion concentration was very low. According to Fig. 4(b), viabilities of EA. hy-926 cells were similar among these three kinds of materials, basically maintained above 90% through the whole test period. However, the viabilities of VSMC decreased as the incubation time increased. After 24 h incubation, the VSMC cells viabilities of all the experimental samples decreased to lower than 60%. This might be attributed to the inhibitory effect of iron ions on the proliferation of VSMC cells^12^.

### Hemolysis of Pt discs patterned pure iron

Figure 4(d) shows the hemolysis percentage of Pt discs patterned pure iron, with uncoated pure iron as control. The hemolysis of Pt discs patterned pure iron was decreased to around 1% when compared to that of uncoated one (approximately 2%). On the whole, the hemolysis of all these materials were lower than 5%, the judging criterion for biomaterials in ASTM F756-08^34^, indicating their good hemocompatibility.

### Platelet adhesion tests on Pt discs patterned pure iron

Figure 4(e) illustrates the number of adhered platelets per unit area on the surface of samples. The number of platelets adhered on the uncoated pure iron was the largest, while that on the Φ4 μm × S4 μm Pt discs patterned pure iron was the least. The morphologies of adhered human platelets on the Pt discs patterned pure iron specimens are shown in Fig. 4(f–h). On the surface of Pt discs patterned pure iron, platelets were more likely to adhere to the pure iron substrate, while it was hard to see platelet adhered on the Pt discs. Of note, large amount of deposited substances were observed. According to the results of previous reports^3^,^23^,^35^,^36^, the deposited substance was mainly composed of corrosion products of iron matrix and salts deposited from PRP and PBS. Most of platelets on all these three kinds of materials were activated and started to extend pseudopodia. Based on the results of platelet adhesion tests, Pt discs patterning can decrease the thrombosis risk of pure iron.

## Discussion

Accelerating degradation rate of pure iron has puzzled scholars for many years. In this study, Pt disc patterns were first adopted to regulate the corrosion behavior of pure iron for biomedical applications. It is widely believed that there are two main methods to accelerate corrosion of pure iron^37^: on the one hand, adding less noble alloying elements into iron matrix to enhance the corrosion tendency; on the other hand, introducing noble metals which can act as cathodes to drive the corrosion of iron matrix (anodes). The standard electrode potential of Pt is +1.2 V, which is much higher than that of Fe (−0.44 V)^38^. In this work, Pt disc arrays on the surface of pure iron was prepared by lithography and electron beam evaporation, aiming at accelerating the degradation rate of pure iron matrix through galvanic corrosion.

**Figure 5:**
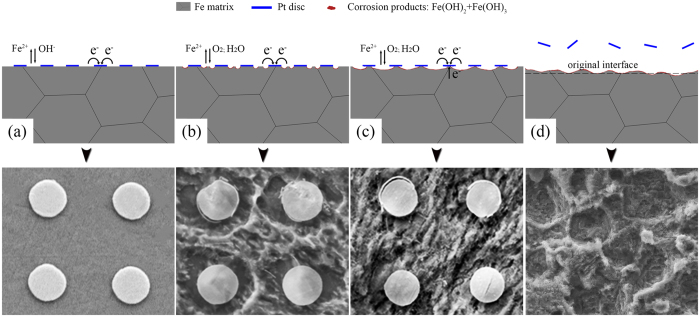
Illustration of the corrosion mechanism for Pt discs patterned pure iron: (**a**) initial corrosion reaction; (**b**) and (**c**) were the formation procedure of hydroxide layer; (**d**) after Pt discs fell off, the degradation rate of pure iron can be continuing accelerated by the corrosion pits.

Figure 5 illustrates the corrosion mechanism of Pt discs patterned pure iron in Hank’s solution. When soaking in Hank’s solution, Pt discs as cathodes formed plenty of galvanic cells with pure iron matrix (anodes), which altered the main corrosion mode of pure iron from localized corrosion into galvanic corrosion. Corrosion first started from the iron matrix closely around Pt discs, which was oxidized into ferrous ions (Equation 1). Electrons generated from iron matrix dissolving were transferred to Pt discs (as shown in Fig. 5(b)) where they were consumed by dissolved oxygen (Equation 2).


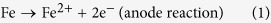



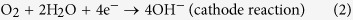


Due to the solution alkalization near Pt discs (Equation 2), iron hydroxide preferentially formed at this place (Equation 3). Since the instability of ferrous hydroxide, it was easy to be oxidized into ferric hydroxide by dissolved oxygen. The reaction can be expressed as Equation 4:


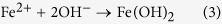



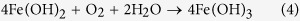


With time, corrosion area gradually expanded into the whole area of exposed iron matrix. Afterwards, corrosion penetrated into the iron matrix beneath the Pt discs as the infiltration of Hank’s solution (Fig. 5(c)). When the whole iron matrix under Pt discs corroded, the Pt discs fell off and corrosion pits remained *in situ*. Consumption of OH^−^ on the tip of corrosion pits formed an acid atmosphere in this region, then pure iron in this region can act as anode. The corrosion current density on this tiny anode can be very large so that pure iron matrix in this region corroded rapidly. Therefore, the depth of corrosion pits increased gradually with time. In addition, as the existence of corrosion pits increased the contacting area of pure iron matrix with corrosive solution, then accelerate the corrosion rate. Therefore, the accelerating effect on degradation of iron matrix can be lasted even after the dropping of Pt discs (Fig. 5(d)).

The phenomenon that the Pt discs patterned pure iron with size of Φ4 μm × S4 μm corroded faster than that with size of Φ20 μm × S5 μm can be explained by theoretical calculations. Pt discs covered corresponding surface area of pure iron, so the sum of area of exposed iron matrix (*A*_*1*_) and Pt discs (*A*_*2*_) should be a constant value ( the area of pure iron before patterning, *A*), which can be expressed as *A*_*1*_ + *A*_*2*_ = *A*. The corrosion current of Pt discs patterned pure iron can be calculated by Equation 5^39^:


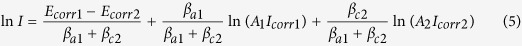


*E*_*corr1*_ and *E*_*corr2*_ are the corrosion potential of Fe and Pt as isolated electrode, respectively. The corresponding corrosion currents are represented as *I*_*corr1*_ and *I*_*corr2*_, respectively. *β*_*a1*_ and *β*_*c1*_ respectively represented the slope of the anode polarization curve and the slope of cathode polarization curve in the natural logarithm Tafel curves of pure iron matrix. *β*_*a2*_ and *β*_*c2*_ respectively represented the slope of the anode polarization curve and the slope of cathode polarization curve in the natural logarithm Tafel curves of Pt discs.

Based on Equation 5, there will be a maximum value of galvanic current with the variation of the area of Pt discs (*A*_*2*_), take the derivative of Equation 5:


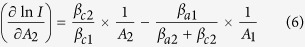


when *A*_*2*_/*A*_*1*_ = *β*_*c2*_/*β*_*a1*_, 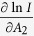 = 0, a maximum value of corrosion current can be obtained.

**Figure 6:**
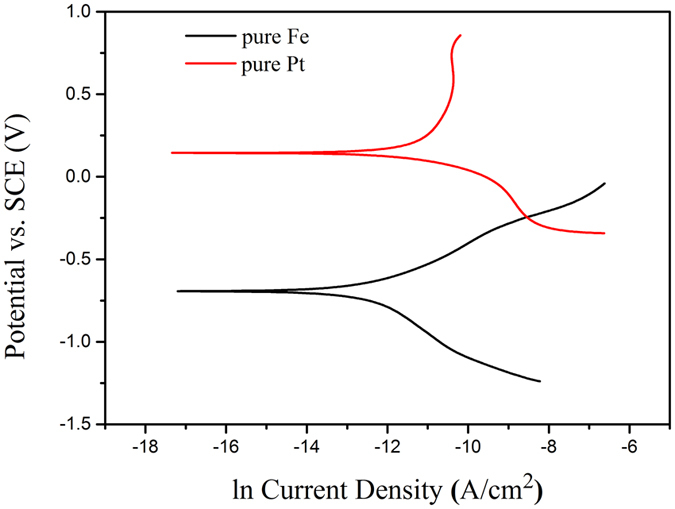
The natural logarithm Tafel curves of pure iron and pure plantinum.

Figure 6 shows the natural logarithm Tafel curves of pure iron and pure platinum measured in Hank’s solution at temperature of 37 ± 0.5 °C. According to these curves, the values of *β*_*a1*_, *β*_*c2*_ can be obtained as 0.399 and 0.102, respectively. Hence, *β*_*c2*_/*β*_*a1*_ = 0.25564 and *A*_*2*_/*A* can be calculated to be 0.2036. That is, when the area of Pt discs occupies 20.36% of the total area, the corrosion current will achieve to its maximum value. By contrast, the bigger gap between 0.2036 and real value of *A*_*2*_/*A*, the smaller the corrosion current is. In terms of Pt discs patterned pure iron with size of Φ4 μm × S4 μm, the value of *A*_*2*_/*A* is 0.19635, which is very close to 0.2036. However, the *A*_*2*_/*A* value of Pt discs patterned pure iron with size of Φ20 μm × S5 μm is 0.50265, which is far away from 0.2036. Therefore, according to above mentioned theoretical calculation, Pt discs patterned pure iron with size of Φ4 μm × S4 μm can corrode faster than the one with size of Φ20 μm × S5 μm.

The corrosion rates of Φ20 μm × S5 μm and Φ4 μm × S4 μm Pt discs patterned pure iron in static immersion test were about 2.33 and 2.58 times as that of pure iron, respectively. Compare to the previous reports^23^,^36^,^40^,^41^ which successfully accelerated corrosion rate of pure iron through various technologies, the corrosion rates of almost all the developed samples in static immersion test were no more than 2 times as that of pure iron, such as Fe-5 wt.% W composite (1.15 times)^23^, Fe-0.5 wt.% CNT composite (1.96 times)^23^ and Fe-5 wt.% Fe_2_O_3_ composite (1.38 times)^36^. There was one exception, the corrosion rate of Fe-5 wt.% Pt composite was about 2.73 times as that of pure iron^33^. However, the high corrosion rate of Fe-5 wt.% Pt composite may be partly due to the low density and the existed FeO.

The biocompatibility of Pt discs patterned pure iron can be discussed from two aspects, including cytotoxicity and hemocompatibility. The cytotoxicity of biodegradable metallic materials are mainly attributed to the released metallic ions^2^ and degradation particles^42^ which can promote or inhibit cell metabolic activities and proliferation. The related studies have shown that Pt ion has significant cytotoxicity^43^. However, the content of Pt ions released into the body fluid would be very low due to the high chemical stability of Pt. The extremely low Pt ion concentration in the extractions measured in this work matched the above deduction. Therefore, the effect of Pt exerted on cells proliferation would be very small. The cytotoxicity of Pt discs patterned pure iron should be mainly attributed to Fe^2+^ and Fe^3+^ ions. According to the work of Zhu^13^, there was almost no effect on vascular endothelial cells when the concentration of iron ions was lower than 50 μg∙mL^−1^. The concentration of iron ions of uncoated pure iron, Φ4 μm × S4 μm and Φ20 μm × S5 μm Pt discs patterned pure iron were 13.601 μg∙mL^−1^, 52.494 μg∙mL^−1^ and 48.784 μg∙mL^−1^, respectively. Although the iron ion concentration was slightly higher than 50 μg∙mL^−1^ for Φ4 μm × S4 μm Pt discs patterned iron, yet no significant cytotoxicity was observed. All the experimental materials showed no obvious toxicity to EA. hy-926 cells. On the contrary, all of these materials strongly hindered the proliferation of vascular smooth muscle cells. Muller *et al*.^12^ found that ferrous ions could exert adverse effect on the proliferation of VSMC cells in the sight of gene expression. According to the report of Schaffer *et al*.^44^, Fe^2+^ and Fe^3+^ ions could repress the migration of smooth muscle cells at the concentration of 1 mM, while good endothelial coverage were found on iron wires.

In terms of hemocompatibility, Pt was confirmed as a very good stent material and has been applied in clinic, such as platinum-iridium alloy stents^45^,^46^,^47^ and platinum-chromium alloy stents^48^. Especially, platinum-iridium alloy stents with the content of Pt over 90 wt.% were applied largely, which showed good effects on the treatment of blood vessel blockages in children congenital heart disease. By this token, Pt is a material with excellent hemocompatibility. From the results of hemolysis tests, pure iron patterned by Pt discs could slightly decrease hemolysis. Moreover, electrochemical corrosion will be triggered when the Pt discs patterned pure iron immersed in serum, the electron released from pure iron matrix (anode) will be transferred to the Pt discs (cathode). Then, the Pt discs are negatively charged. The main cells in blood, including erythrocyte, leukocyte and platelet, are also negatively charged. Therefore, the negatively charged Pt discs might reject the platelets adhesion by electrostatic interaction. Previous works had also demonstrated that negatively charged materials can prevent platelet adhesion^49^,^50^. Before the adhesion of platelets, plasma proteins, including albumin, globin and fibrin, are competitively absorbed on the surface of biomaterials. The most abundant platelet membrane component glycoprotein IIb/IIIa is the receptor of Arg-Gly-Asp (RGD) peptide on fibrin^51^. Therefore, the absorption of fibrin plays a dominant role to promote the adhesion of platelet. However, the albumin is an anticoagulant protein which can hinder the adhesion of platelets and leucocytes^52^. Negatively charged surface can enhance the absorption of albumin but causes an adverse impact on absorption of fibrin^53^. Therefore it can be speculated that the negatively charged platinum discs in the present study would prevent platelets adhesion.

Then the number of adhered platelets on the surface of Pt discs patterned pure iron was much less than that on the surface of uncoated pure iron. All these results proved that Pt patterning has the potential to decrease the risk of thrombosis of pure iron.

In summary, two different designs of platinum disc arrays, including sizes of Φ20 μm × S5 μm and Φ4 μm × S4 μm, have been coated on the surface of pure iron in this work. The influence of Pt disc arrays on the degradation of pure iron matrix *in vitro* was investigated by electrochemical tests and static immersion tests. The results indicated that coating platinum disc arrays on the surface of pure iron can form a plenty of galvanic cells to accelerate the degradation of pure iron. Simultaneously, due to the designability of the shape, size as well as distribution of Pt discs, the degradation rate as well as uniformity of pure iron can be effectively controlled by coating with platinum discs. The *in vitro* biocompatibility of Pt discs patterned pure iron has also been researched by indirect cytotoxicity tests, hemolysis tests and platelet adhesion tests. Pt discs patterned pure iron exhibited almost no toxicity to human umbilical vein endothelial cells (EA. hy-926), but performed a significant inhibition on proliferation of vascular smooth muscle cells (VSMC). In addition, the hemolysis of Pt discs patterned pure iron was lower than 1%. Moreover, the number of adhered platelets on pure iron coated with platinum discs was less than that on uncoated pure iron. Especially for pure iron coated by Pt discs with size of Φ4 μm × S4 μm, the number of adhered platelets was the lowest. These results indicated pure iron patterned by platinum discs have the potential to decrease the risk of thrombosis.

## Materials and Methods

### Materials preparation

**Figure 7:**
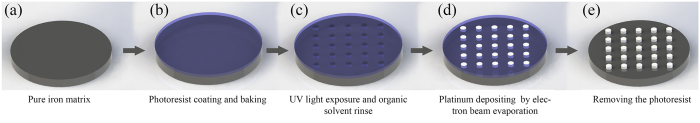
The process of preparation of Pt discs patterned pure iron.

Two different designs of platinum disc arrays, including sizes of Φ20 μm × S5 μm and Φ4 μm × S4 μm, have been deposited on the surface of pure iron (99.95% purity, ZhongNuo Advanced Material (Beijing) Technology Co., China) through photolithography and electron beam evaporation. Figure 7 illustrates the preparation procedure. First of all, a layer of photoresist (Shipley-9912) was spin coated on the polished pure iron (Φ75 × 1 mm^2^), then covered by a lithography mask. Afterwards, specimens were exposed to ultraviolet light and the part without the protection of lithography mask was rinsed in organic solvent (4 wt.% NaOH solution). After rinsing, a layer of photoresist with via-hole arrays was obtained. Electron beam evaporation was adopted to deposit Pt (99.99% purity, ZhongNuo Advanced Material (Beijing) Technology Co., China) on the surface of pure iron across the via-holes. Lastly, photoresist layer was removed using acetone.

### Microstructure characterization

Pt disc arrays on the surface of pure iron was observed using environmental scanning electron microscope (ESEM, Quanta 200FEG), with an energy dispersive spectrometer (EDS) attached. The energy dispersive spectrometer was employed for the analysis of chemical composition.

### Electrochemical measurements

Electrochemical measurements were carried out using a traditional three-electrode cell at an electrochemical work station (PGSTAT 302 N, Metrohm Autolab). The specimen, a saturated calomel electrode (SCE) and a platinum electrode were acted as working electrode, reference electrode and the auxiliary electrode, respectively. All the measurements were maintained at temperature of 37 ± 0.5 °C in Hank’s solution^54^ with pH value of 7.4. The area of working electrode exposed to the solution was 0.3318 cm^2^. The open circuit potential (OCP) measurement was set for 9000 s. The potentiodynamic polarization curves were carried out from (OCP value − 600) mV (vs. SCE) to (OCP value + 600) mV (vs. SCE) at a scanning rate of 0.33 mV·s^−1^. An average of at least three measurements was taken for each group. The corrosion rates were calculated according to ASTM-G102-89^55^ using the following formulas:


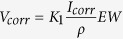



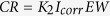


where *V*_*corr*_ (mm/yr) is the corrosion rate in terms of penetration rate, CR (mg·cm^−2^·day^−1^) is the corrosion rate in terms of mass loss rate, *EW* is the equivalent weight, *K*_*1*_ = 3.27 × 10^−3^ mm·g·μA^−1^·cm^−1^·year^−1^ and *K*_*2*_ = 8.954 × 10^−3^ g·cm^2^·μA^−1^·m^−2^·day^−1^.

### Static immersion tests

Static immersion tests were performed in Hank’s solution for 3, 7, 14, 28 and 42 days, respectively. 50 mL Hank’s solution was used for each sample following ASTM-G31-72^56^ at 37 °C in water bath. After 3, 7, 14, 28 and 42 days respectively, the samples were removed from the solution, gently rinsed with distilled water and quickly dried in case of oxidation. Changes on the surface morphologies after immersion were characterized by ESEM (Quanta 200FEG). Samples were immersed in the 10 mol/L NaOH solution to remove the corrosion products before weighing. An average of three measurements was taken for each group. The degradation rates were calculated based on the formula below:


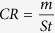


where *CR* (mg·cm^−2^·day^−1^) is the corrosion rate, *m* (mg) is the mass loss, *S* (cm^2^) is the surface area of the specimen exposed to the solution and *t* (day) is the immersion time.

### Cytotoxicity tests

The cytotoxicity tests were performed by indirect contact methods. Human umbilical vein endothelial cells (EA. hy-926) and human vascular smooth muscle cells (VSMC) were used to evaluate the cytotoxicity of the Pt discs patterned pure iron. At first, cells were cultured in the Dulbecco’s modified Eagle’s medium (DMEM) with 10% fetal bovine serum (FBS), 100 U·mL^−1^ penicillin and 100 μg·mL^−1^ streptomycin at 37 °C in a humidified atmosphere with 5% CO_2_. According to ISO 10993-12^57^, extraction medium was prepared using serum-free DMEM with a surface area/extraction medium ratio of 1.25 cm^2^·mL^−1^ in a humidified atmosphere with 5% CO_2_ at 37 °C for 72 h. After the extracts were centrifuged, the supernatant fluid was withdrawn and stored at 4 °C before cytotoxicity test. The control groups involved DMEM medium as the negative control and DMEM including 10% dimethyl sulfoxide (DMSO) as the positive control. The concentrations of metallic ions in the extraction medium were measured by inductively coupled plasma atomic emission spectrometry (ICP-AES) (Leeman, Profile). Cells were incubated in the 96-well plates at the density of approximately 5 × 10^3^ cells per 100 μL medium in each well and incubated for 24 h to allow attachment. DMEM was then replaced by extraction mediums, and 10 μL serum was added to each well. After 1, 2 and 4 days incubation, 10 μL of cell counting kit (CCK-8) solution was added to each well, then continued incubating for 3 h. The absorbance of each well was tested using microplate reader (Bio-RAD680) at the wavelength of 450 nm. Viability of cells (X) was calculated using the following formula according to ISO 19003-5^58^:


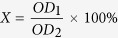


Here *OD*_*1*_ is the mean absorbance of experimental sample groups and positive control group. *OD*_*2*_ is the mean absorbance of negative control group.

### Hemolysis test and platelet adhesion

Healthy human blood (anticoagulant was 3.8 wt.% citric acid sodium) extracted from volunteers was diluted by physiological saline based on volume ratio of 4:5. Uncoated pure iron and Pt discs patterned pure iron were separately put into centrifugal tubes with 10 mL physiological saline for 30 minutes, temperature was kept at 37 °C. Then 0.2 mL diluted blood was added to each tube and incubated at 37 °C for 60 minutes, 10 mL deionized water with 0.2 mL diluted blood as the positive control and 10 mL physiological saline with 0.2 mL diluted blood as the negative control. After completion of the above steps, samples were removed, and then these tubes were centrifuged at 800 g for 5 minutes. Supernatant was transferred to 96-well plates, the absorbance (OD) was determined by a microplate reader (Bio-RAD680) at the wavelength of 545 nm. Hemolysis of samples was calculated by the following formula:


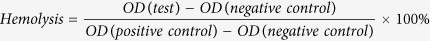


For platelet adhesion, whole blood from healthy human body was centrifuged at 1000 r/min for 10 minutes. Platelet rich plasma (PRP) was obtained from the upper fluid. Samples after ultraviolet disinfection were moved to 24-well plates and 0.2 ml PRP was added to each well, then incubated at 37 °C for 1 h. After gently rinsed by phosphate buffered saline (PBS), platelets on samples were fixed using 2.5% glutaraldehyde solution at room temperature for 1 h. Then dehydrated with gradient alcohol solution (concentration of 50%, 60%, 70%, 80%, 90%, 95% and 100%), each concentration for 10 min, and finally freeze-dried for 2 days. The morphologies of platelet adhered on the specimens were observed by ESEM (Quanta 200FEG).

### Statistical analysis

All quantitative data are expressed as mean ± standard deviations with n = 3. Statistical analysis was performed by one way analysis of variance (ANOVA) followed by Tukey’s post hoc tests using SPSS 19.0 and p-values less than 0.05 were considered statistically significant.

## Additional Information

**How to cite this article**: Huang, T. and Zheng, Y. Uniform and accelerated degradation of pure iron patterned by Pt disc arrays. *Sci. Rep.* **6**, 23627; doi: 10.1038/srep23627 (2016).

## Figures and Tables

**Table 1:** Average electrochemical parameters of Pt discs patterned pure iron (uncoated pure iron as control).

Materials	*E*_*corr*_ (V)	*I*_*corr*_(μA·cm^−2^)	*V*_*corr*_(mm/year)	Corrosion rate (mg·cm^−2^·d^−1^)	
				Electrochemical test	Immersion test for 42 days
Uncoated pure iron	−0.69932	9.64230	0.11204	0.24127	0.14853
Φ4 μm × S4 μm	−0.88616	19.754	0.22256	0.47927	0.38324
Φ20 μm × S5 μm	−0.76282	17.698	0.20565	0.44285	0.34565

Note: Corrosion potential (*E*_*corr*_), corrosion current density (*I*_*corr*_) and corrosion rate (*V*_*corr*_).
